# Expression of the zinc finger transcription factor Sp6–9 in the velvet worm *Euperipatoides kanangrensis* suggests a conserved role in appendage development in Panarthropoda

**DOI:** 10.1007/s00427-020-00661-w

**Published:** 2020-05-19

**Authors:** Ralf Janssen, Graham E. Budd

**Affiliations:** Department of Earth Sciences, Uppsala University, Palaeobiology, Villavägen 16, Uppsala, Sweden

**Keywords:** Arthropod development, Appendage development, Sp1, SP5, Buttonhead, Panarthropoda, Onychophora

## Abstract

The Sp-family genes encode important transcription factors in animal development. Here we investigate the embryonic expression patterns of the complete set of Sp-genes in the velvet worm *Euperipatoides kanangrensis* (Onychophora), with a special focus on the *Sp6–9* ortholog. In arthropods, *Sp6–9*, the ortholog of the *Drosophila melanogaster D-Sp1* gene plays a conserved role in appendage development. Our data show that the expression of *Sp6–9* during the development of the velvet worm is conserved, suggesting that the key function of the *Sp6–9* gene dates back to at least the last common ancestor of arthropods and onychophorans and thus likely the last common ancestor of Panarthropoda.

**Electronic supplementary material:**

The online version of this article (10.1007/s00427-020-00661-w) contains supplementary material, which is available to authorized users.

## Introduction

Sp-family genes encode a conserved group of transcription factors, all of which possess three highly conserved C_2_H_2_-type zinc fingers that bind to G-rich regions in their target genes and a so-called buttonhead box (reviewed in, e.g. Kadonaga et al. 1987; Suske 1999; Kolell and Crawford 2002; Schaeper et al. 2010). The name “Sp” originates from the technique used to first purify an “SP” gene using Sephacryl columns and phosphocellulose chromatography (Dynan and Tjian 1983). It has been shown that Sp genes bind to a plethora of target genes and are thus involved in many developmental processes (reviewed in, e.g. Suske et al. 2005). The last common ancestor of all metazoans already possessed three Sp genes (Schaeper et al. 2010). In vertebrates, however, gene duplication has led to a much larger complement of Sp genes, and since much of the initial research on Sp genes comes from vertebrates, the nomenclature of Sp genes may to some degree be confusing. Sp genes are named Sp1 to Sp9. Sp1 to Sp4 are related and form the first clade (Sp1–4), Sp5 is the only member of its own clade, and Sp6 to Sp9 form the third clade (Sp6–9) (Suske et al. 2005; Zhao and Meng 2005; Schaeper et al. 2010; Suske 2017).

In arthropods, the situation is much simpler: there is one gene representing each class, Sp1–4, Sp5 and Sp6–9 (Schaeper et al. 2010). The first Sp gene to be identified and studied in the model arthropod *Drosophila melanogaster* was *buttonhead* (*btd*), which represents the mammalian Sp5 ortholog (Wimmer et al. 1993). The single arthropod member of the Sp1–Sp4 clade (Sp1–4) (*CG5669* in *Drosophila*) is expressed ubiquitously and at equal levels during development, and it may thus represent a universal transcription factor (summarized in Schaeper et al. 2010). The third arthropod Sp gene is the single ortholog of the Sp6 to Sp9 clade (Sp6–9) (called *D-Sp1* in *Drosophila*) (Wimmer et al. 1996). *Drosophila btd* (*Sp5*) and *D-Sp1* (*Sp6–9*) are expressed in similar patterns in post-blastoderm stages, and it has been shown that the two genes can at least partially substitute for each another (Wimmer et al. 1996; Schöck et al. 1999). Subsequent research, however, has shown that it is *Sp6–9* that represents a key factor of appendage development in *Drosophila*, a function that is not fully shared by *Sp5/btd* (Estella and Mann 2010; Cordoba et al. 2016). Functional studies in *Drosophila* (Cordoba et al. 2016) and other arthropods including sequentially segmenting insects such as the red flour beetle *Tribolium castaneum* (Beermann et al. 2004), the true bug *Oncopeltus fasciatus* (Schaeper et al. 2009) and the spider *Parasteatoda tepidariorum* (Königsmann et al. 2017; Setton and Sharma 2018) showed that limb growth is heavily disturbed in a *Sp6–9* depleted or knock-down background, suggesting that the role of *Sp6–9* as a key factor in limb growth is conserved among Arthropoda as a whole.

In this paper, we present the gene expression patterns of the three Sp genes, *Sp1–4*, *Sp5/buttonhead-like* (*Sp5/btdl*) and *Sp6–9* in the onychophoran *Euperipatoides kanangrensis*. Onychophorans are closely related to arthropods and may indeed represent their sister group (e.g. Campbell et al. 2011), although the relationship of Onychophora, Tardigrada and Arthropoda is not fully resolved yet (reviewed in Giribet and Edgecombe 2017).

The data show that the role of *Sp6–9* in appendage growth is likely conserved in onychophorans and thus likely in all of Panarthropoda. The expression of the onychophoran *Sp5/btdl* ortholog suggests a certain degree of redundancy of the more closely related Sp genes *Sp6–9* and *Sp5/btdl* (Schaeper et al. 2010), as reported for *D-Sp1* (*Sp6–9*) and *btd* (*Sp5*) in *Drosophila* (Wimmer et al. 1996; Schöck et al. 1999). As in arthropods, the onychophoran *Sp5/btdl* gene may play an early role during germ band patterning, albeit not, unlike in arthropods, restricted to head development. Unlike the situation in arthropods, the onychophoran *Sp1–4* gene is not expressed ubiquitously but superficially resembles the patterns of *Sp5/btd* and *Sp6–9*.

## Methods

### Animal husbandry and fixation of embryos

Embryos were obtained and treated for subsequent in situ hybridization experiments as described in Hogvall et al. (2014). Developmental stages are described in Janssen and Budd (2013).

### Phylogenetic analysis

Sp-family genes were identified performing reciprocal BLAST searches against the sequenced embryonic transcriptome of *Euperipatoides* using the sequences of *Drosophila* sp. orthologs as baits.

Amino acid sequences of the conserved regions of putative Sp-genes, and the Cabut protein sequence of *Drosophila* that serves as an outgroup sequence, were aligned using T-Coffee followed by manual editing in SeaView (Notredame et al. 2000; Gouy et al. 2010) using default parameters as suggested for MacVector v12.6.0 (MacVector, Inc., Cary, NC). A Bayesian phylogenetic analysis was executed in MrBayes (Huelsenbeck and Ronquist 2001) with a fixed WAG amino acid substitution model with gamma-distributed rate variation across sites (with four rate categories), unconstrained exponential prior probability distribution on branch lengths and exponential prior for the gamma shape parameters for among site rate variation. The tree topology was calculated applying 500,000 cycles for the Metropolis-Coupled Markov Chain Monte Carlo (MCMCMC) analysis (four chains; chain-heating temperature of 0.2). Markov chains were sampled every 200 cycles. Default settings were used, defining 25% of the samples as burn-in information. Clade support was calculated with posterior probabilities in MrBayes. Unique sequence identifiers of all sequences used in the analysis are listed in Supplementary File F1. The nexus file and the alignment are available as Supplementary Files F2 and F3.

### Gene cloning, whole mount in situ hybridization and nuclear staining

Sections of SP-genes were amplified by RT-PCR with gene-specific primers that were based on the information from the sequenced embryonic transcriptome of *Euperipatoides* (PRJNA525753: SRR8690378). For all genes, nested PCRs were run with internal primers, using 1 μl of the product of a first PCR as template. Primer sequences are listed in Supplementary File F4. All gene fragments were cloned into the PCRII vector (Invitrogen) and sequenced on an ABI3730XL automatic sequencer (Macrogen, Seoul, South Korea). Gene-identifying numbers are summarized in Supplementary File F1. In situ hybridizations were performed using a universally working protocol that is described in Janssen et al. (2018, supplement). Cell nuclei were stained with 1:10000 SYBR-Green (Invitrogen) in phosphate buffered saline with 0.1% Tween-20 (PBST-0.1%) for approximately 20 min at room temperature.

### Data documentation

Bright-field microscopy and visualization of SYBR-Green stain were performed with a Leica-DC490 digital camera that was equipped with a UV light source mounted onto a MZ-FLIII Leica dissection microscope. Linear adjustments of colour contrast and brightness were executed using the image-processing software Adobe Photoshop CS6 for Apple Macintosh (Adobe Systems Inc.)

## Results and discussion

### Sequence analysis

**Fig. 1:**
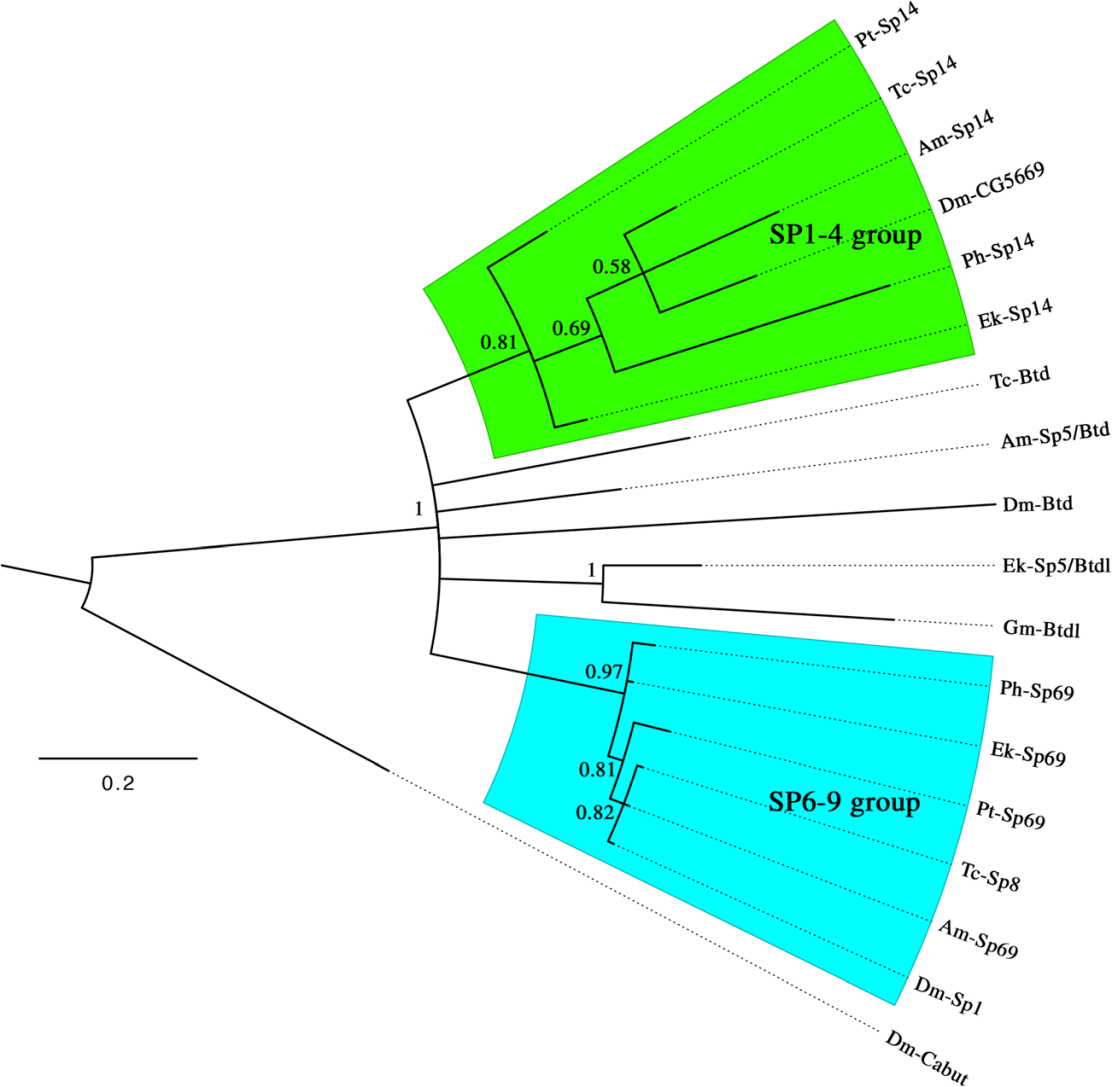
Phylogenetic analysis of Dmrt genes. Species abbreviations: Am, *Apis mellifera* (Hexapoda/Hymenoptera); Ek, *Euperipatoides kanangrensis* (Onychophora); Dm, *Drosophila melanogaster* (Hexapoda/Diptera); Gm, *Glomeris marginata* (Myriapoda/Diplopoda); Ph, *Parhyale hawaiensis* (Pancrustacea/Amphipoda); Pt, *Parasteatoda tepidariorum* (Chelicerata/Aranea); Sm, *Strigamia maritima* (Myriapoda/Chilopoda). Green shade: Sp1–4 group. Blue shade: Sp6–9 group. Node support is given as posterior probabilities. Note that support for the Sp1–4 group and the Sp6–9 group is high, but Sp5/Btd-like factors do not form a clear monophyletic group

Three Sp-family genes were identified in *Euperipatoides* (Fig. 1) suggesting that onychophorans possess the full complement of Sp genes (Schaeper et al. 2010). As in other phylogenetic analyses (Schaeper et al. 2009, 2010; Königsmann et al. 2017), members of the Sp1–4 and Sp6–9 clade cluster with high confidence, while members of the more-derived buttonhead (Btd) and Sp5 clade often cluster with low support or remain unresolved, as in this study (Fig. 1). The onychophoran *Sp5/btdl* sequence, however, clusters with the previously described *btd*-like (*btdl*) sequence from the myriapod *Glomeris marginata* (Janssen et al. 2011). In some arthropod species, especially chelicerates, *Sp5 and btd*-like genes appear to have been frequently lost or are difficult to recognize (Setton and Sharma 2018). This may be the reason why the *btd*-like gene escaped from recognition in an earlier analysis of gap and head gap gene-like genes in *Euperipatoides* (Janssen 2017a).

### Sp6–9 genes are conserved factors of appendage development in panarthropods

**Fig. 2:**
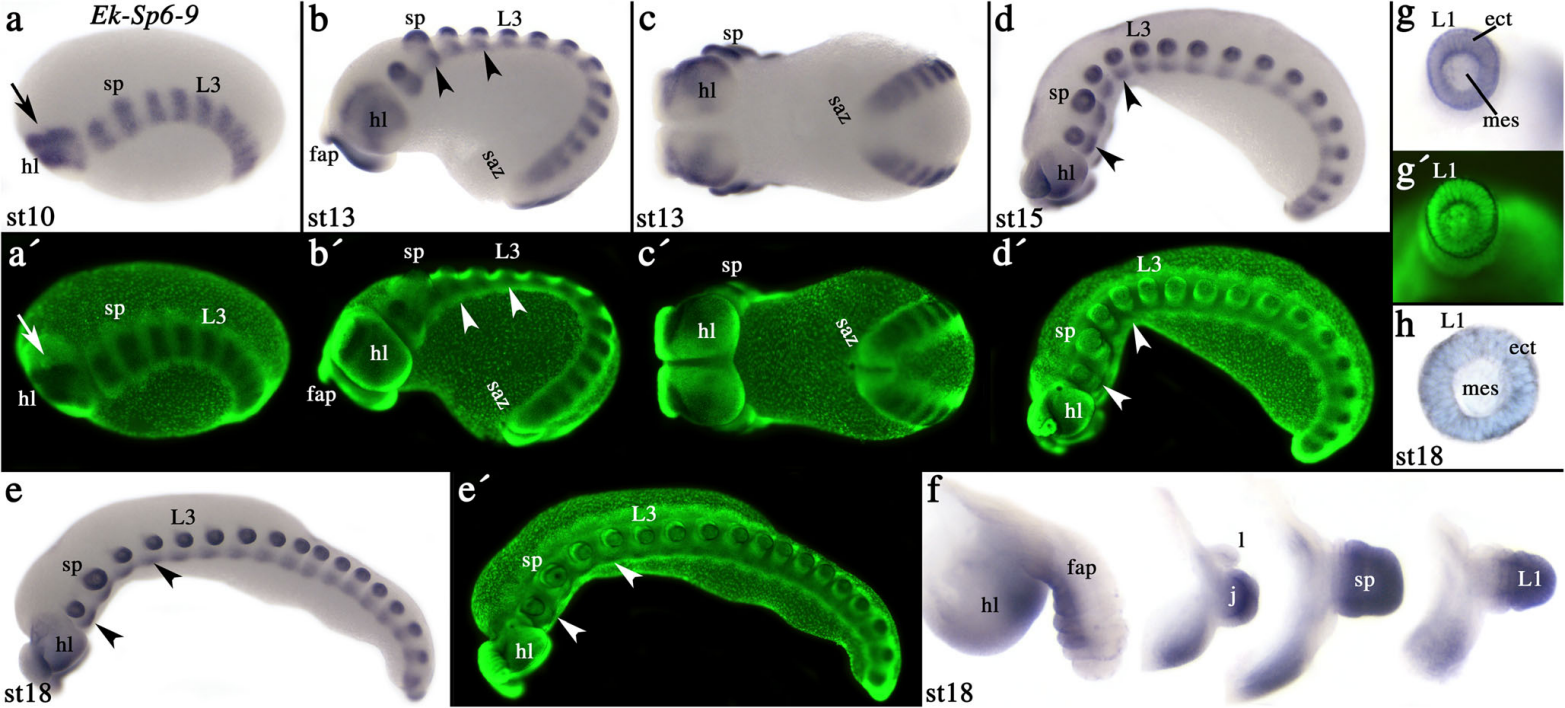
Expression of *Euperipatoides Sp6–9*. In all panels, except panels (**F**) and (**G**), anterior is to the left. Developmental stages are indicated. **A** Lateral view. The arrow points to the frontal appendage. **B** Lateral-ventral view. Arrowheads point to expression in the ventral nervous system. **C** Same embryo as in (**B**), ventral view. **D** and **E** Lateral views on later stage embryos. Arrowheads as in panel (**C**). **F** Dissected appendages. **G** On top view of a walking leg with removed tip. Anterior down. Note that expression is in the ectoderm, but not the mesoderm. **H** On top view of a dissected walking leg with removed tip and base. The photograph shows a bright-field picture of a SYBR-Green counter-stained leg. **A´**–**E´** and **G´** represent SYBR-Green counter-staining of the corresponding bright-field pictures. Abbreviations: ect, ectoderm; fap, frontal appendage; hl, head lobe; l, lip; L, walking limb; mes, mesoderm; saz, segment addition zone; and sp, slime papilla

In *Euperipatoides*, *Sp6–9* is initially expressed in the brain that develops within the paired head lobes and in the most anterior trunk segments (Fig. S1). Within this domain, expression is stronger at the position where the appendages will form, but tissue between these limb primordia initially also expresses *Sp6–9*, albeit not so strongly (Fig. S1). The posterior segment addition zone (SAZ) and the last formed posterior segments do not express *Sp6–9* (Fig. S1). Later, expression between the limb primordia disappears, resulting in a segmental pattern along the AP body axis in the regions where the limbs form (jaws, slime papillae and legs) (Fig. 2A). At subsequent developmental stages, this expression splits into a domain in the outgrowing limbs and expression in the tissue ventral to the base of the limbs that is likely contributing to the developing ventral nervous system (Fig. 2B, D, E).

In the growing appendages (Fig. 2B–F), expression is restricted to their distal ectoderm (Fig. 2G, H), while the proximal region does not express *Sp6–9* (Fig. 2F). This overall expression pattern persists throughout development. !While the expression in the jaws, the slime papillae and the legs is restricted to the distal region, in the frontal appendages (the onychophoran antennae), the most anterior pair of appendages, the pattern is different. Here, *Sp6–9* is expressed along the ventral ectoderm with exception of the very tip (Fig. 2A–F). Expression of *Sp6–9* in all appendages, except the frontal appendages, is virtually identical with the expression of *Sp6–9* orthologs in other arthropods including *Drosophila* (Wimmer et al. 1996; Schaeper et al. 2009, 2010; Königsmann et al. 2017; Setton and Sharma 2018) and thus in line with a general role in limb growth.

The finding that the arthropod appendage-patterning key factor *Sp6–9* is expressed in conserved patterns in the onychophoran is not surprising given that also other conserved factors of arthropod appendage development such as *Distal-less* (*Dll*), *dachshund* (*dac*) and *homothorax* (*hth*) are likely conserved in onychophorans (Angelini and Kaufman 2005; Janssen et al. 2010, 2015; Pechmann et al. 2010; Oliveira et al. 2014; Heingård et al. 2019). Altogether, these data indicate that the gene regulatory network orchestrating appendage growth is conserved in Panarthropoda.

The different expression of *Sp6–9* in the frontal appendages (antennae; albeit not homologous with the antennae of Pancrustacea (e.g. Eriksson et al. 2010)) is remarkable. The frontal appendages are either considered to have evolved independently from the other appendages or represent highly derived serially homologous appendages, possibly homologous with the labrum of arthropods (reviewed in Ortega-Hernández et al. 2017). If the former is true, expression patterns may be generally different, although conserved genetic networks may have been recruited for its development. If the latter is true, expression in the frontal appendages is best compared with the arthropod labrum, which is dorso-ventrally reversed due to rotation and fusion of these appendages in the lineage leading to Arthropoda (Kimm and Prpic 2006). Thus, genes that are expressed ventrally in the labrum are expressed dorsally in the other appendages and vice versa.

Despite the fact that *Sp6–9* has been investigated in a wide range of arthropod species, expression in the labrum has unfortunately not been in the focus of these studies (Schaeper et al. 2009, 2010; Königsmann et al. 2017; Setton and Sharma 2018). In all hitherto investigated arthropod species, however, *Sp6–9* is expressed in the labrum, and it appears that this expression is dorsal. We confirmed this by taking a closer look at the expression of *Sp6–9* in the spider *Parasteatoda tepidariorum* (Fig. S2).

The dorsal expression of *Sp6–9* in the arthropod labrum and the ventral expression in the frontal appendage of onychophorans are thus conserved, supporting their possible homology. The overall gene expression profile and presence (or absence) of genes in the developing onychophoran frontal appendages and the labrum of arthropods, however, is not universally conserved (Janssen 2017b).

### Sp5/btd-like genes

Embryonic expression of the onychophoran *Sp5/btdl* gene is in some aspects similar to that of *Sp6–9*. Unlike *Sp6–9*, however, *Sp5/btdl* is expressed very early during development in tissue around the blastopore (posterior pit) and the forming mouth-anus furrow (Fig. S3A, B), but note that the lips of the blastopore do not express *Sp5/btdl* (Fig. S3). When the furrow expands along the AP axis of the embryo, *Sp5/btdl* first remains expressed in its lips and in the tissue between the mouth-anus furrow and the embryo proper (Fig. S3C, D). Later, *Sp5/btdl* disappears from this latter tissue and from the anterior of the mouth-anus furrow (the later mouth) (cf. Janssen et al. 2015)) (Fig. S3E); expression in the posterior of the furrow, the later anus, however, remains (Fig. S3E–G).

**Fig. 3:**
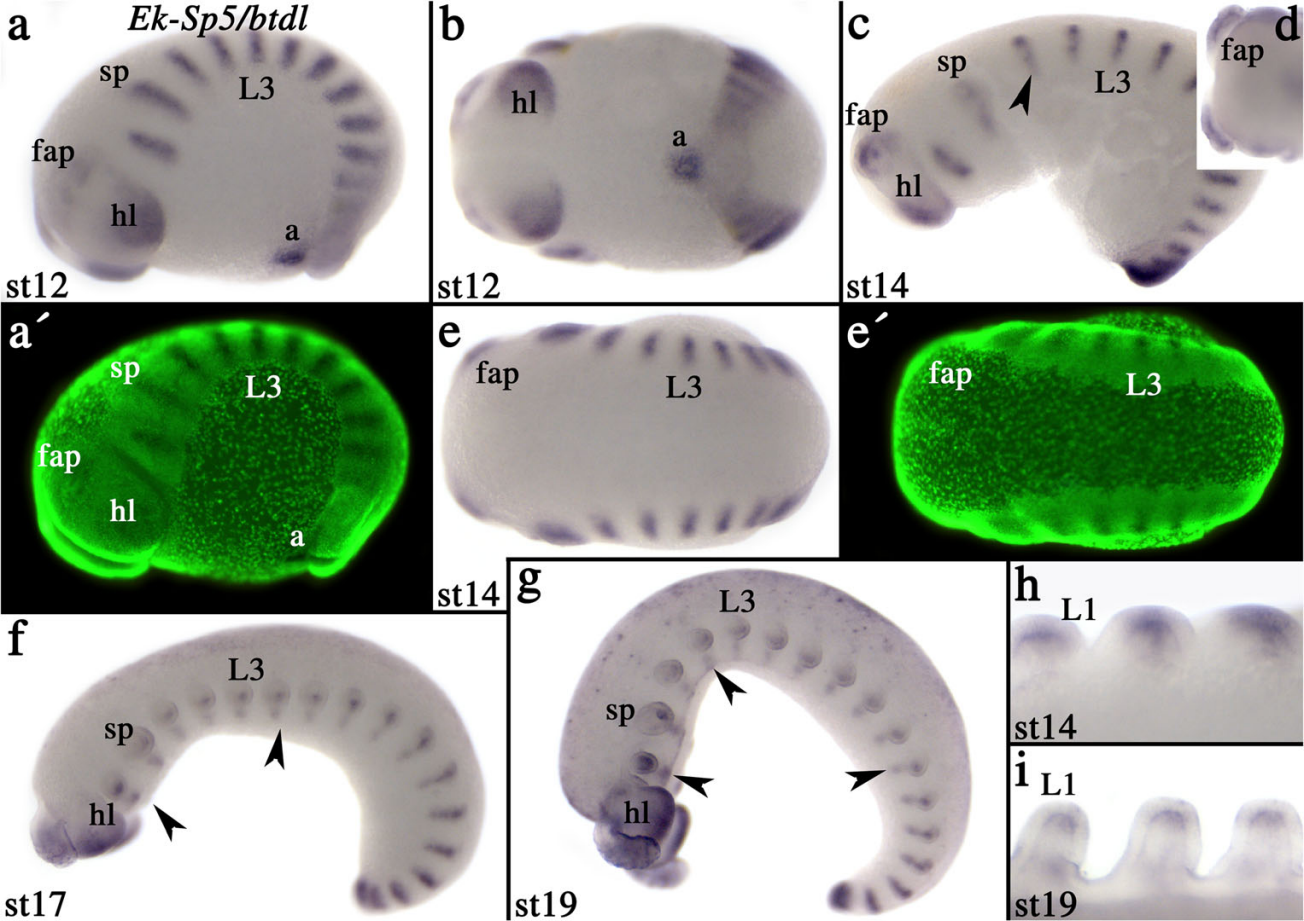
Expression of *Euperipatoides Sp5/btdl*. In all panels, anterior is to the left. Developmental stages are indicated. **A** Lateral view. Note the segmental segmentation gene-like pattern of *Sp5/btdl*. **B** Same embryo as in (**A**), ventral view. **C** Lateral view. The arrowhead points to expression in the ventral nervous system. **D** Dorsal view on anterior of embryo shown in (**C**). **E** Dorsal view of embryo shown in (**C**). **F** and **G** Lateral views on later-stage embryos. Arrowheads as in panel (**C**). **H** and **I** Ventral view on walking legs. Note expression in the tips of the legs. **A´** and **E´** represent SYBR-Green counter-staining of the corresponding bright-field pictures. Abbreviations as in Fig. 2, a, anus

From stage 8 onwards, expression refines into transverse segmental stripes and a differentiated pattern in the posterior of the head lobes; the anterior of the head lobes remains free from expression (Fig. S3E–G and Fig. 3A, B). In contrast to *Sp6–9*, these segmental stripes are thinner. After limb growth begins, the stripes resolve into a pattern in the tips of the appendages and the ventral nervous system (much like for *Sp6–9*) (Fig. 3C, F, G). Expression in the appendages is restricted to the tips (Fig. 3D–I).

The expression profile of *Euperipatoides Sp5/btdl* is consistent with a role in the brain and ventral nervous system development as well as appendage development, each of which are characteristics of arthropod *Sp5/btdl* genes (e.g. Estella et al. 2003; Schinko et al. 2008; Schaeper et al. 2010). Unlike in at least mandibulate arthropods where *Sp5/btdl* is expressed early during development in a head gap gene-like domain (Wimmer et al. 1993; Schinko et al. 2008; Schaeper et al. 2010; Janssen et al. 2011; Hunnekuhl and Akam 2017; Jeon et al. 2019), and the gap gene function of *Sp6/9* in a spider (Königsmann et al. 2017; Setton and Sharma 2018), there is no such pattern in the onychophoran that would suggest a similar function as a gap gene, neither of *Sp6/9* nor of *Sp5/btd* (Supplementary Figs. S1 and S3). This may be little surprising given that the gap-gene-like network as known from *Drosophila* and as partially conserved in other arthropods (reviewed in, e.g. Damen 2007; Jaeger 2011) is not conserved in onychophorans (Franke and Mayer 2015; Janssen 2017a, b). What the early function of *Sp5/btdl* in onychophorans is, however, remains unclear.

### Sp1–4 genes

Data on arthropod Sp1–4 genes are restricted to the analysis of expression patterns. One reason for this may be the fact that hitherto investigated arthropod Sp1–4 genes are expressed ubiquitously during ontogenesis (summarized in Schaeper et al. 2010) and may thus represent universal transcription factors or may be regulated post-transcriptionally.

**Fig. 4:**
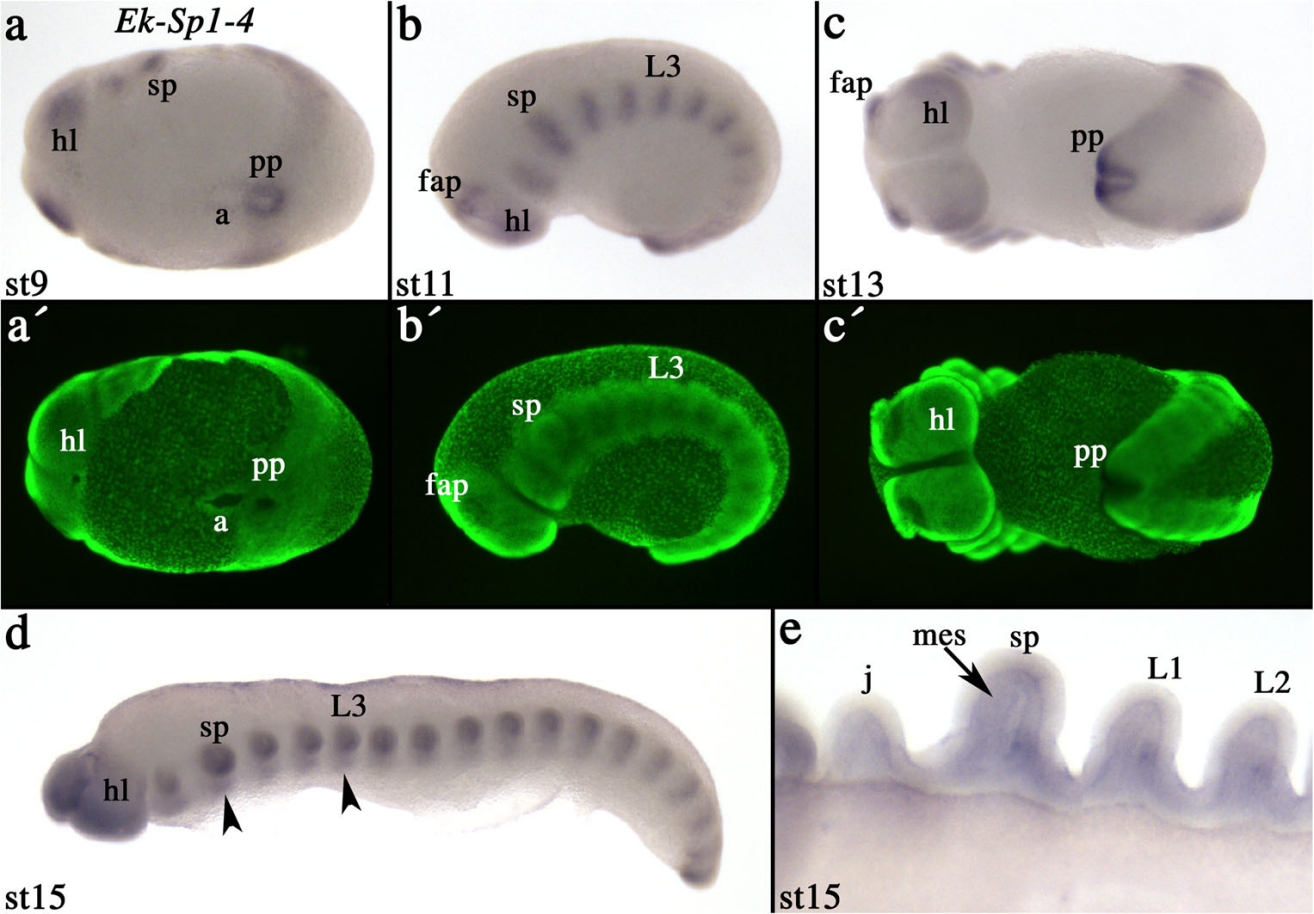
Expression of *Euperipatoides Sp1–4*. In all panels, anterior is to the left. Developmental stages are indicated. **A** Ventral view. **B** Lateral view. Note the segmentation-gene like expression of *Sp1–4*. **C** Ventral view. Note the strong expression in the posterior pit region. **D** Lateral view. Arrowheads point to expression in the ventral nervous system. **E** Close-up on the appendages of the embryo shown in (**D**). Ventral view. The arrow points to expression in the mesoderm. **A´–C**´ represent SYBR-Green counter-staining of the corresponding bright-field pictures. Abbreviations as in Fig. 2, a, anus; pp, posterior pit

In the onychophoran, however, *Sp1–4* is not expressed ubiquitously but rather in a pattern that is similar to that of the other two Sp genes (cf. Figs. 2, 3, and 4). Specifically, *Euperipatoides Sp1–4* is expressed in the brain, but not in exactly the same pattern as either *Sp5/btdl* or *Sp6–9* (Fig. 4A–C), and as for *Sp5/btdl* and *Sp6–9*, in the form of transverse segmental stripes that later during development split into a domain in the limbs and a domain in the developing ventral nervous system (Fig. 4D/E). Unlike *Sp5/btdl* and *Sp6–9*, *Sp1–4* is also expressed in the posterior pit that may suggest a function in segment addition and/or posterior elongation (Fig. 4A/C). Expression in the developing appendages is mainly restricted to the mesoderm (Fig. 4E).

## Electronic supplementary material

ESM 1 (DOCX 42 kb)

ESM 2 (NEX 2 kb)

ESM 3 (MSAP 22 kb)

ESM 4 (DOCX 39 kb)

Supplementary Figure S1 Early expression of Euperipatoides Sp6-9PanelsA and Brepresent ventral views, panel C represents a ventral-lateral view. All embryos are oriented with their anterior to the left. Developmental stages are indicated.Arrowheads point to expression between the primordia of the limbs; note that at later developmental stages, this expression will disappear. See main text for further information. A ´-C ´ represent SYBR-Green staining of the embryos shown in A-C. hl, head lobe; j, jaw bearing segment; L1, first walking leg bearing segment; sp, slime papilla bearing segment (PNG 1214 kb)

High resolution image (TIF 30769 kb)

Supplementary Figure S2 Expression of Sp6-9 in the labrum of the spider Parasteatoda tepidariorumA. Anterior up. Flat-mounted head of a stage 11 embryo.BAnterior to the left. Stage 10.2 embryo. B ´ SYBR-Green stained embryo as shown in B. Abbreviations: br, brain; ch, chelicera; lr, labrum. (PNG 1070 kb)

High resolution image (TIF 23842 kb)

Supplementary Figure S3 Early expression of Euperipatoides Sp5/btdlAll panels represent ventral views and embryos are oriented with their anterior to the left. Developmental stages are indicated. In all panels, arrows point to the blastopore (the posterior pit) that does not express Sp5/btdl. Arrowheads point to expression in the mouth-anus furrow at earliest developmental stages (A-D), and later in the posteriorof the mouth-anus furrow that will contribute to the anus (E-G).The barsin panels E and E ´ mark the anterior region of the mouth-anus furrow that does no longerexpress Sp5/btdlat this stage.See main text for further information. Panels A ´-C ´ and E ´ represent SYBR-Green stained embryos as seen in panels A-C, and E. Abbreviations: (hl) most anterior of the split germ band that will later form the head lobes; hl, head lobe; sp, slime papilla bearing segment; L3, third walking limb bearing segment. (PNG 2515 kb)

High resolution image (TIF 60425 kb)
