# Supplementary material for: Expression of the zinc finger transcription factor Sp6–9 in the velvet worm Euperipatoides kanangrensis suggests a conserved role in appendage development in Panarthropoda
Source: Dev Genes Evol. 2020 May 19;230(3):239–45. doi: 10.1007/s00427-020-00661-w (PMC7260272; doi:10.1007/s00427-020-00661-w)
Supplement: Supplementary file 1 — (DOCX 42 kb) [file 427_2020_661_MOESM1_ESM.docx]

Am-Sp1-4 XP_006564631.1

Am-Sp5/Btd XP_001119912.1

Am-Sp6-9 XP_012341200.1

Dm-Btd NP_511100.1

Dm-Sp1 NP_727360.1

Dm-Sp1-4/CG5669 ABA86490.1

Dm-Cabut NP_722636.1

Ek-Sp1-4 c205105_g1_i3

Ek-Sp5/Btd c202137_g1_i1

Ek-6-9 c214633_g3_i1

Gm-Btdl CAK50835

Ph-Sp1-4 CBH30980.1

Ph-Sp6-9 AXP83337.1

Pt-Sp1-4 AVT42523.1

Pt-Sp6-9 MG857620.1

Tc-Sp1-4 XP_015833716.1

Tc-Sp5/Btd NP_001107792.1

Tc-Sp8 NP_001034509.1
