# Supplementary material for: Expression of the zinc finger transcription factor Sp6–9 in the velvet worm Euperipatoides kanangrensis suggests a conserved role in appendage development in Panarthropoda
Source: Dev Genes Evol. 2020 May 19;230(3):239–45. doi: 10.1007/s00427-020-00661-w (PMC7260272; doi:10.1007/s00427-020-00661-w)
Supplement: Supplementary file 4 — (DOCX 39 kb) [file 427_2020_661_MOESM4_ESM.docx]

Ek_SP14_c205105_FW1 GGAGGAAGTTATCAACGGGA

Ek_SP14_c205105_FW2 TATCGTCCAAGTTCCGCAAG

Ek_SP14_c205105_BW1 GGTTTGAACTGTTTGCACCG

Ek_SP14_c205105_BW2 TGTGCGTTGACCATAGTACC

Ek_SP5/btdl_c202137_FW1 AACAAGCAATCTCCTCTGGC

Ek_SP5/btdl_c202137_FW2 CATGGTCAAATCTCTGCCTC

Ek_SP5/btdl_c202137_BW1 GTTCAGTTGTTAGGAGCAAG

Ek_SP5/btdl_c202137_BW2 CTATCAACGTCTACTTGCTC

Ek_SP69_c214633_FW1 GGCAAGCTCTTTAATTGGGG

Ek_SP69_c214633_FW2 GCACTCCTCTAGCGATGTTA

Ek_SP69_c214633_BW1 ATCACCATCAGTTACAGGCG

Ek_SP69_c214633_BW2 GTGGCTCAAAGGTGGTGTAT
