# Supplementary figures and images for: Expression of the zinc finger transcription factor Sp6–9 in the velvet worm Euperipatoides kanangrensis suggests a conserved role in appendage development in Panarthropoda
Source: Dev Genes Evol. 2020 May 19;230(3):239–45. doi: 10.1007/s00427-020-00661-w (PMC7260272; doi:10.1007/s00427-020-00661-w)

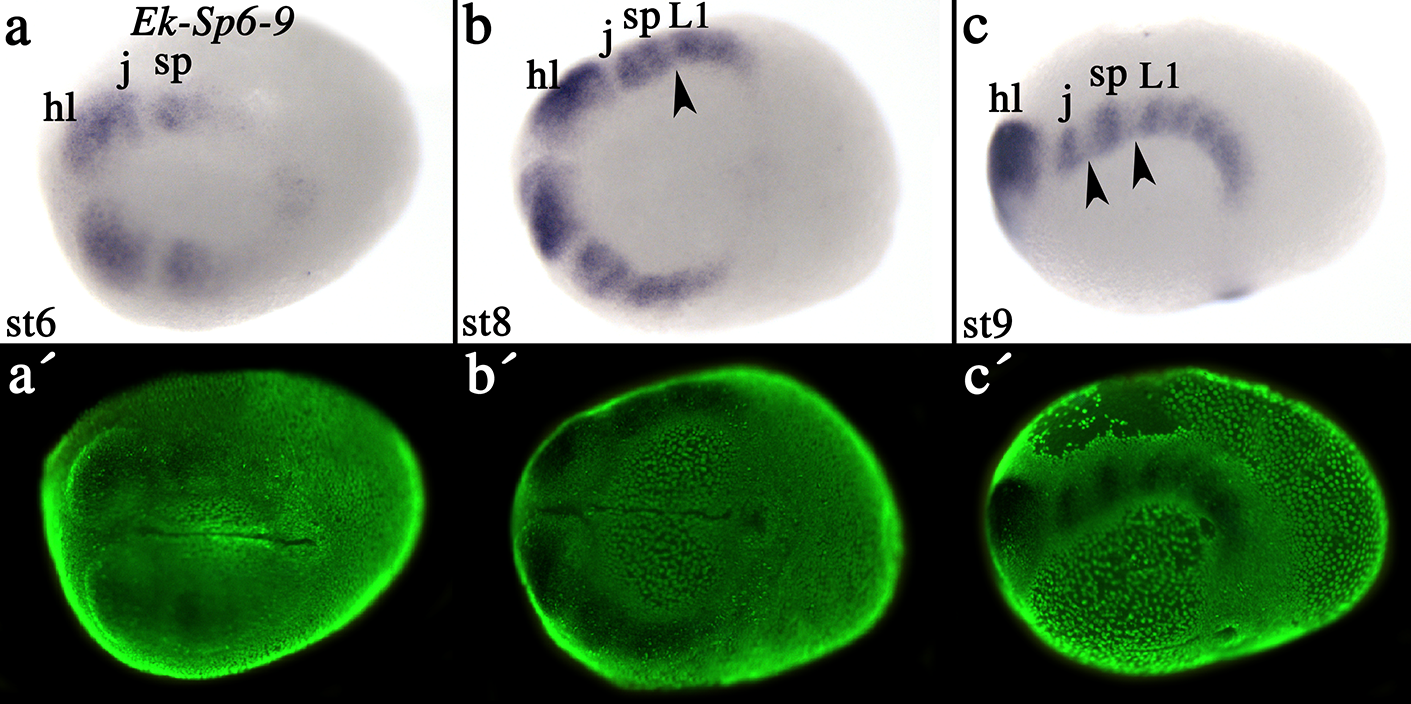

Supplement: Supplementary file 5 — Early expression of Euperipatoides Sp6-9PanelsA and Brepresent ventral views, panel C represents a ventral-lateral view. All embryos are oriented with their anterior to the left. Developmental stages are indicated.Arrowheads point to expression between the primordia of the limbs; note that at later developmental stages, this expression will disappear. See main text for further information. A ´-C ´ represent SYBR-Green staining of the embryos shown in A-C. hl, head lobe; j, jaw bearing segment; L1, first walking leg bearing segment; sp, slime papilla bearing segment (PNG 1214 kb) [file 427_2020_661_Fig5_ESM.png]

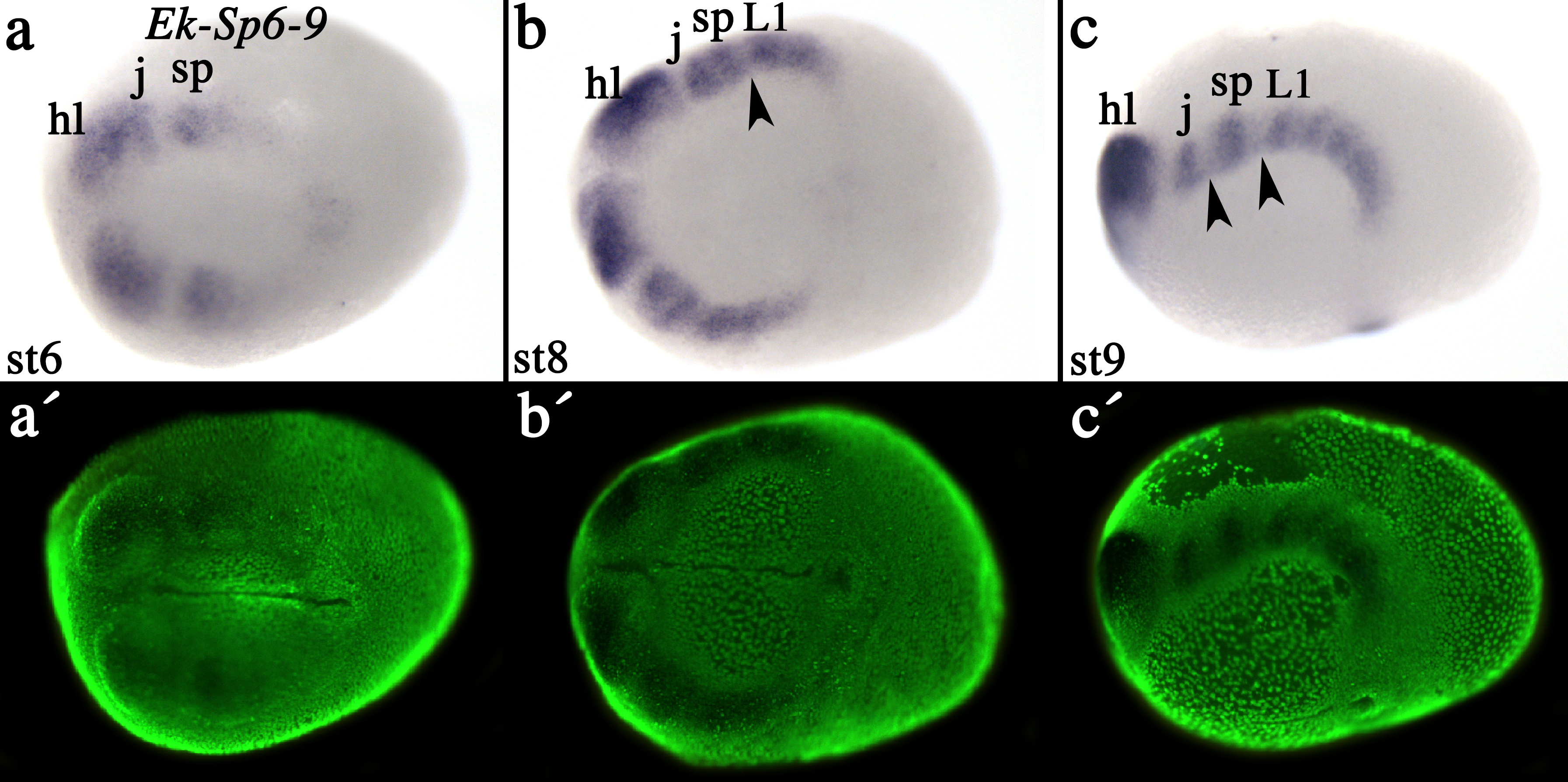

Supplement: Supplementary file 6 — High resolution image (TIF 30769 kb) [file 427_2020_661_MOESM5_ESM.tif]

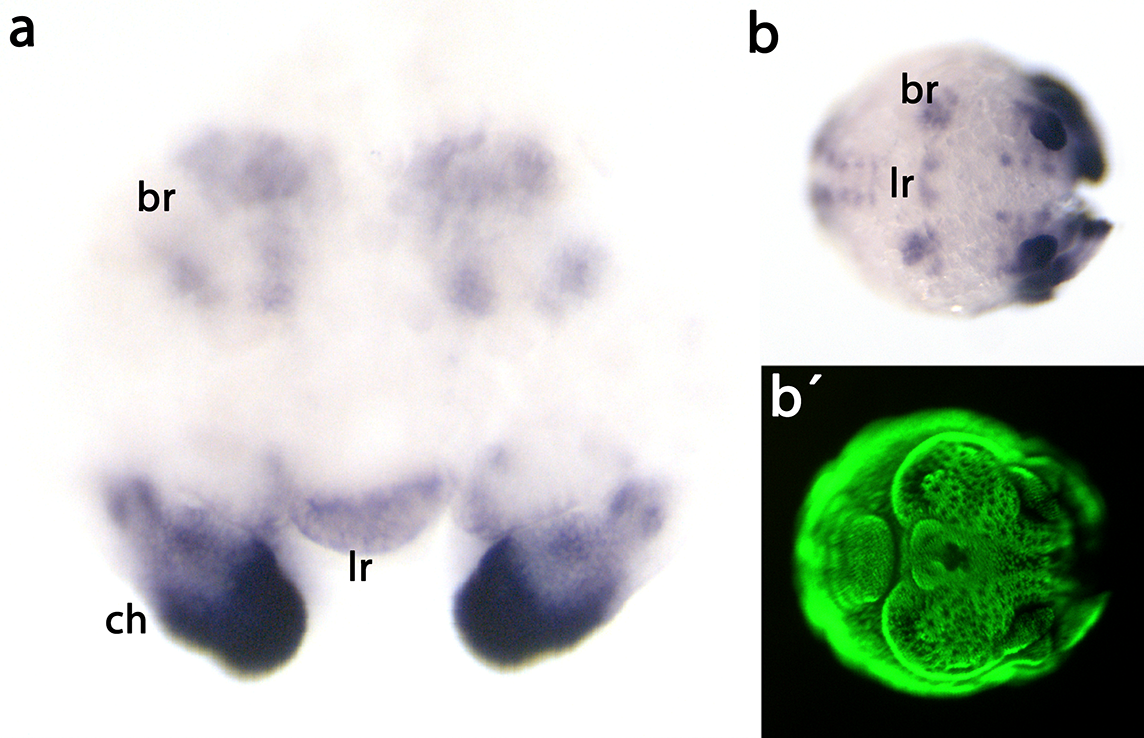

Supplement: Supplementary file 7 — Expression of Sp6-9 in the labrum of the spider Parasteatoda tepidariorumA. Anterior up. Flat-mounted head of a stage 11 embryo.BAnterior to the left. Stage 10.2 embryo. B ´ SYBR-Green stained embryo as shown in B. Abbreviations: br, brain; ch, chelicera; lr, labrum. (PNG 1070 kb) [file 427_2020_661_Fig6_ESM.png]

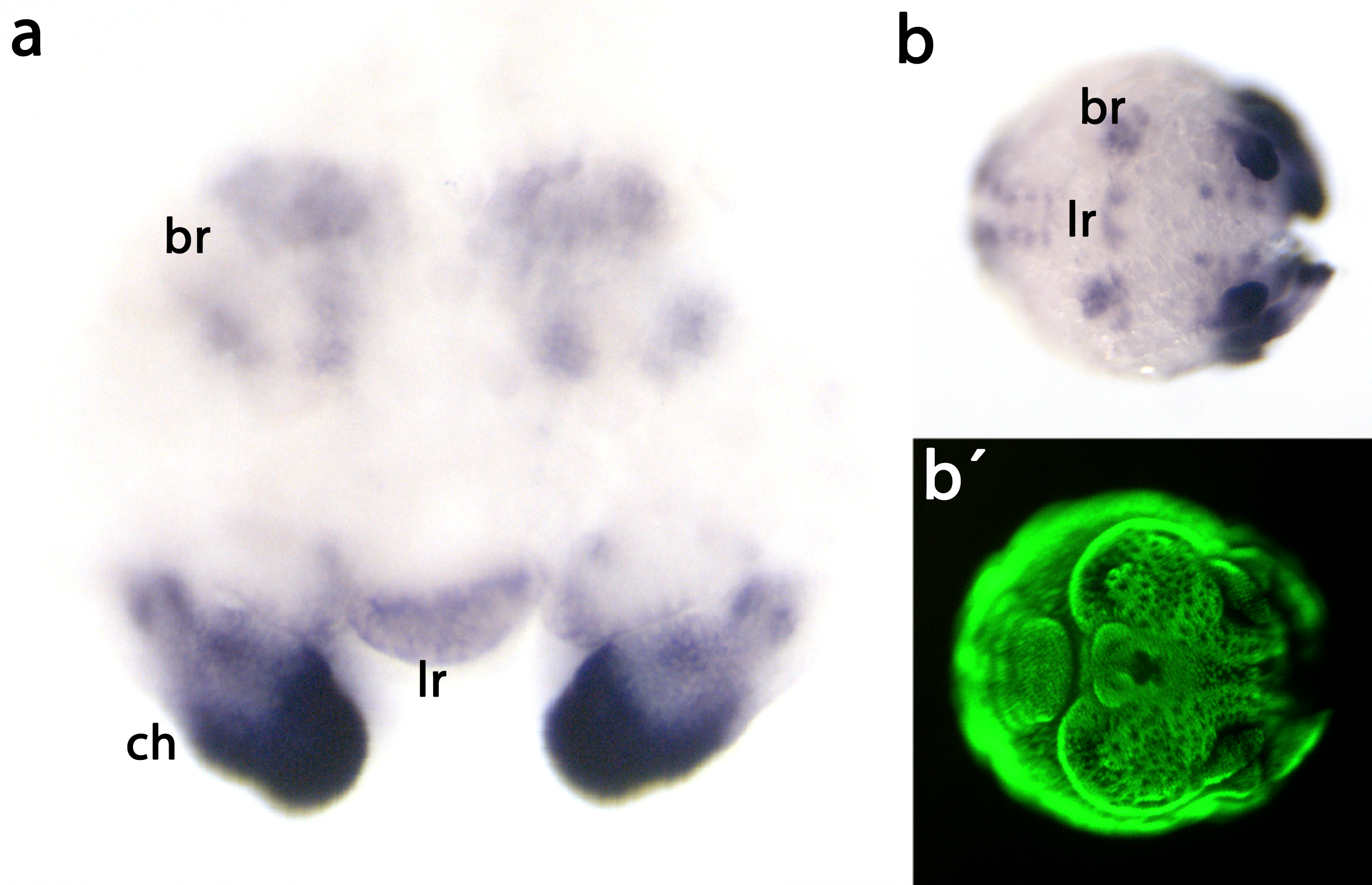

Supplement: Supplementary file 8 — High resolution image (TIF 23842 kb) [file 427_2020_661_MOESM6_ESM.tif]

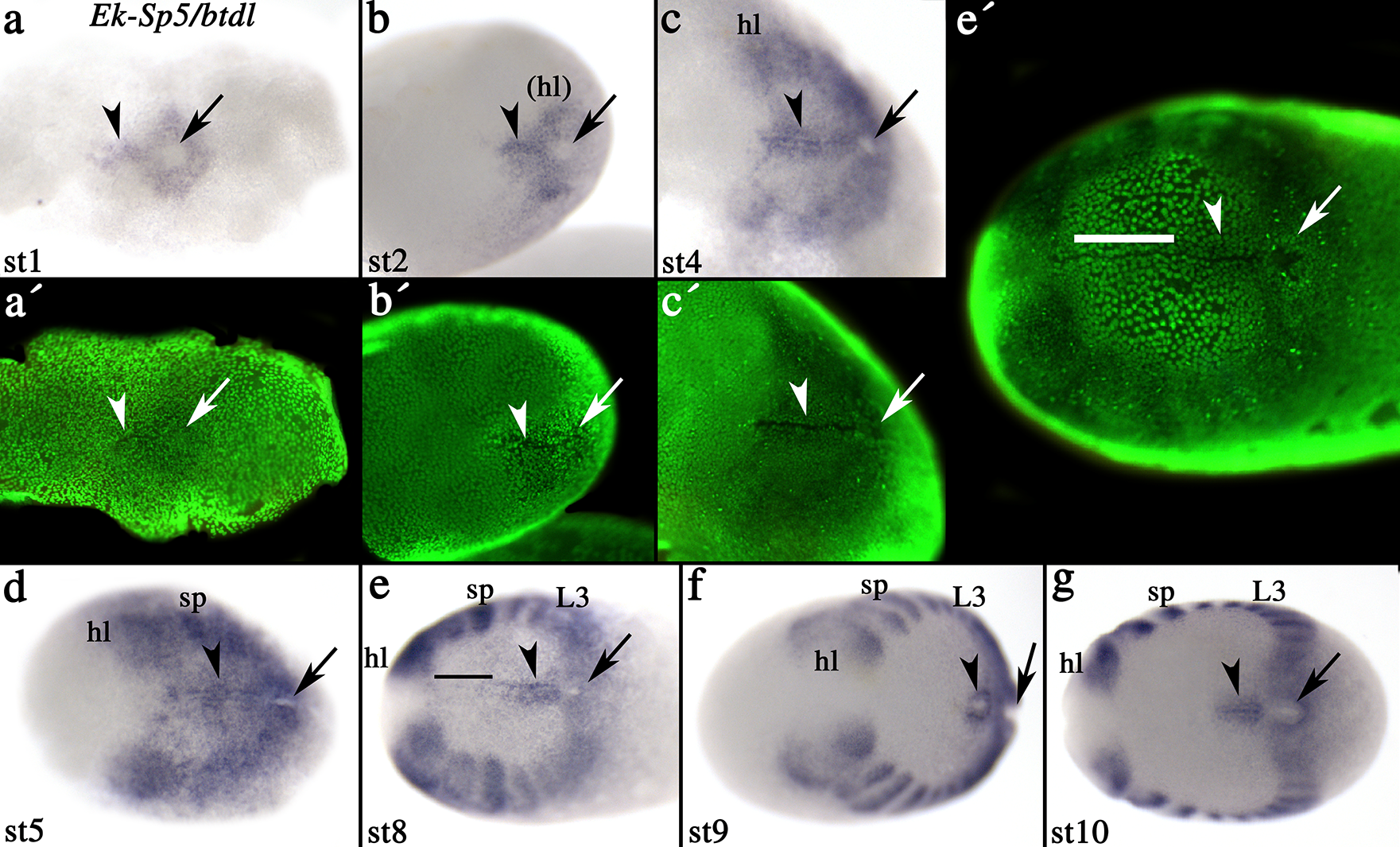

Supplement: Supplementary file 9 — Early expression of Euperipatoides Sp5/btdlAll panels represent ventral views and embryos are oriented with their anterior to the left. Developmental stages are indicated. In all panels, arrows point to the blastopore (the posterior pit) that does not express Sp5/btdl. Arrowheads point to expression in the mouth-anus furrow at earliest developmental stages (A-D), and later in the posteriorof the mouth-anus furrow that will contribute to the anus (E-G).The barsin panels E and E ´ mark the anterior region of the mouth-anus furrow that does no longerexpress Sp5/btdlat this stage.See main text for further information. Panels A ´-C ´ and E ´ represent SYBR-Green stained embryos as seen in panels A-C, and E. Abbreviations: (hl) most anterior of the split germ band that will later form the head lobes; hl, head lobe; sp, slime papilla bearing segment; L3, third walking limb bearing segment. (PNG 2515 kb) [file 427_2020_661_Fig7_ESM.png]

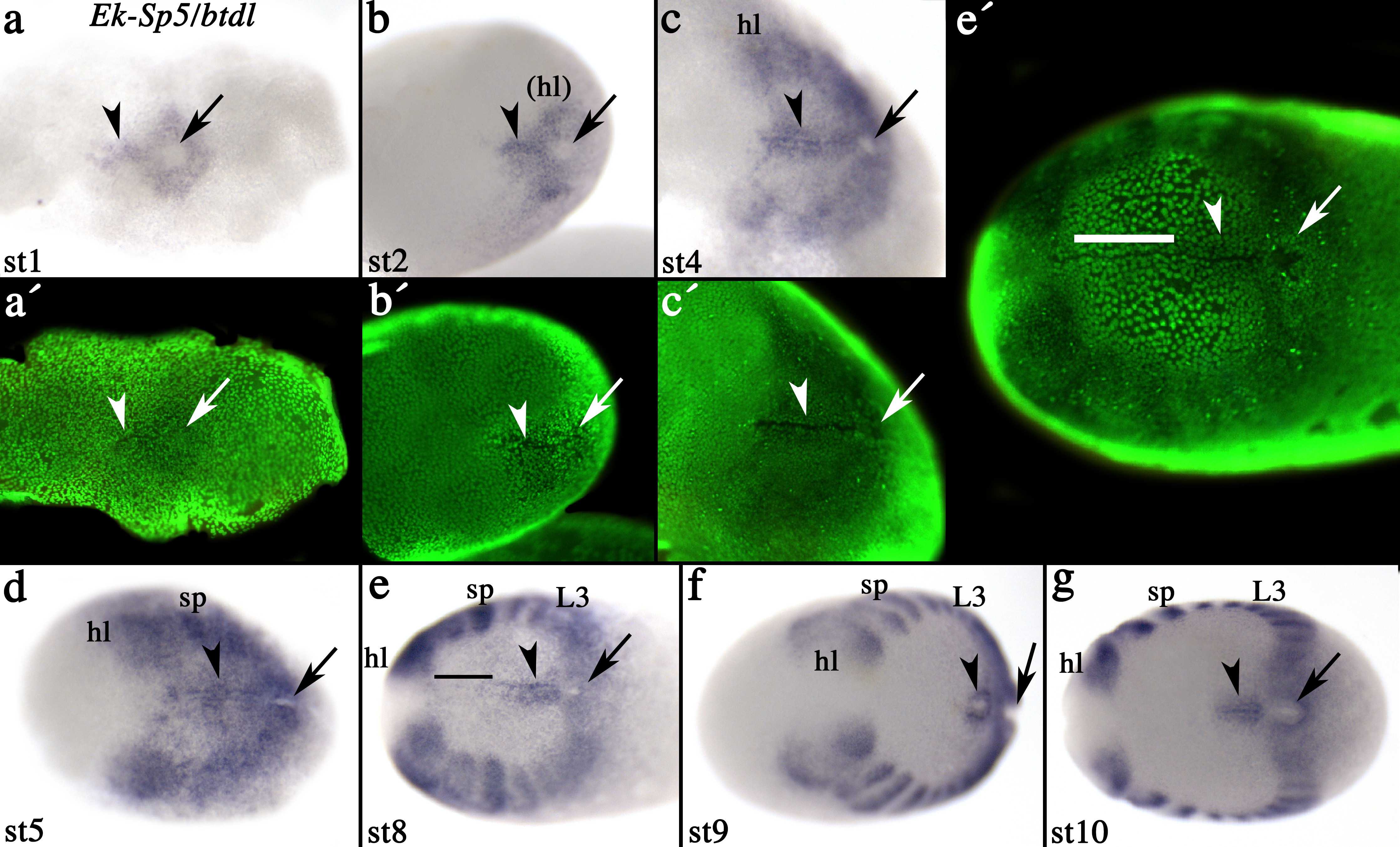

Supplement: Supplementary file 10 — High resolution image (TIF 60425 kb) [file 427_2020_661_MOESM7_ESM.tif]
